# Little Evidence of Antagonistic Selection in the Evolutionary Strata of Fungal Mating-Type Chromosomes (*Microbotryum lychnidis-dioicae)*

**DOI:** 10.1534/g3.119.400242

**Published:** 2019-04-23

**Authors:** Anna Liza Bazzicalupo, Fantin Carpentier, Sarah Perin Otto, Tatiana Giraud

**Affiliations:** * Department of Botany, 3200-6270 University Blvd., University of British Columbia, Vancouver, BC V6T 1Z4, Canada; † Ecologie Systématique Evolution, Univ. Paris-Sud, CNRS, AgroParisTech, Université Paris-Saclay, 91400 Orsay, France; ‡ Department of Zoology & Biodiversity Research Centre, 6270 University Blvd., University of British Columbia, Vancouver, BC V6T 1Z4, Canada

**Keywords:** antagonistic selection, fungi, mating-type chromosomes, evolutionary strata, expression, sex chromosomes, sexual antagonism, haploid selection, Genetics of Sex

## Abstract

Recombination suppression on sex chromosomes often extends in a stepwise manner, generating evolutionary strata of differentiation between sex chromosomes. Sexual antagonism is a widely accepted explanation for evolutionary strata, postulating that sets of genes beneficial in only one sex are successively linked to the sex-determining locus. The anther-smut fungus *Microbotryum lychnidis-dioicae* has mating-type chromosomes with evolutionary strata, only some of which link mating-type genes. Male and female roles are non-existent in this fungus, but mating-type antagonistic selection can also generate evolutionary strata, although the life cycle of the fungus suggests it should be restricted to few traits. Here, we tested the hypothesis that mating-type antagonism may have triggered recombination suppression beyond mating-type genes in *M. lychnidis-dioicae* by searching for footprints of antagonistic selection in evolutionary strata not linking mating-type loci. We found that these evolutionary strata (i) were not enriched in genes upregulated in the haploid phase, where cells are of alternative mating types, (ii) carried no gene differentially expressed between mating types, and (iii) carried no genes displaying footprints of specialization in terms of protein sequences (d_N_/d_S_) between mating types after recommended filtering. Without filtering, eleven genes showed signs of positive selection in the strata not linking mating-type genes, which constituted an enrichment compared to autosomes, but their functions were not obviously involved in antagonistic selection. Thus, we found no strong evidence that antagonistic selection has contributed to extending recombination suppression beyond mating-type genes. Alternative hypotheses should therefore be explored to improve our understanding of the sex-related chromosome evolution.

Recombination between different genotypes can generate beneficial allelic combinations and purge deleterious mutations (Otto 2009). Paradoxically, the genomic regions involved in promoting such genetic exchanges are often substantially excluded from these benefits, having evolved recombination suppression (Idnurm *et al.* 2015). Examples of such genomic regions include the sex chromosomes determining male and female phenotypes in many plants and animals, self-incompatibility loci in plants, and mating-type loci in fungi (Uyenoyama 2005; Beukeboom and Perrin 2014; Idnurm *et al.* 2015; Charlesworth 2016). The suppression of recombination in these genomic regions maintains the allelic combinations required for correct sex or mating-type determinism by linking, for example, pheromone and pheromone receptor alleles. This represents a case of beneficial allelic associations of multiple genes through linkage, more generally called “supergenes” (Charlesworth 2016; Branco *et al.* 2018). On sex-related chromosomes (considered generically to include chromosomes bearing mating-type genes, as in the *Microbotryum* fungus studied here), recombination suppression often extends well beyond the genes involved in sex determination, which is puzzling, given the reduced efficiency of selection in the absence of recombination (Marais *et al.* 2008; Otto 2009). The lack of recombination is consequently thought to lead to the accumulation of deleterious alleles through Muller’s ratchet (Charlesworth and Charlesworth 1997; Immler and Otto 2015) and contribute to the degeneration of the non-recombining chromosome in many species (*e.g.*, Marais *et al.* 2008; Otto 2009). Extensive differentiation between the non-recombining sex chromosomes (*e.g.*, between the X and Y) often arises through a series of recombination suppression steps. With each round of recombination suppression, an “evolutionary stratum” is generated, with the amount of genetic differentiation between the sex chromosomes within the region recording the time since the cessation of genetic exchange.

Understanding the nature of selection underlying the stepwise differentiation of sex chromosomes is a field of active research in evolutionary biology (Beukeboom and Perrin 2014; Wright *et al.* 2016). Sexually antagonistic selection, in which selection favors different alleles in the two sexes, is thought to be a particularly important driver of sex chromosome differentiation and recombination suppression (Bergero and Charlesworth 2009; Charlesworth 2016; Wright *et al.* 2016). However, empirical support for the hypothesis that sexually antagonistic genes accumulate near sex-determining regions, causing the successive steps of recombination suppression for their linkage to sex-determining loci, is slim, despite numerous studies in diverse plant and animal systems (Beukeboom and Perrin 2014; Wright *et al.* 2016). This lack of support may reflect the challenge of identifying sexually antagonistic loci (*e.g.*, Kasimatis *et al.* 2017; Mank 2017) and/or the relative lack of studies focusing on young sex chromosomes, where the signals of selection are not yet affected by degeneration.

Alternative hypotheses have also been put forward to explain the progressive expansion of recombination suppression between the sex chromosomes (Ironside 2010; Ponnikas *et al.* 2018). This may involve other forms of selection besides sexually antagonistic selection, such as conflicting selection pressures between haploid and diploid phases (Scott and Otto 2017) and heterozygote advantage (Antonovics and Abrams 2004; Otto 2014 ; Immler and Otto 2015). In a recent study on *Rumex*, for example, sex-linked genes were found to be more highly expressed in the haploid (pollen) phase, suggesting that conflicts between haploid and diploid phases may drive the evolution of sex chromosomes (Sandler *et al.* 2018). In inbreeding organisms, like the *Microbotryum* fungus studied here, linkage to the sex- or mating-type-determining region is a potent mechanism to preserve heterozygosity, which can favor recombination suppression (Charlesworth and Wall 1999). On the other hand, such inbreeding restricts the conditions under which a polymorphism can be maintained (*e.g.*, Charlesworth and Wall 1999), suggesting that selectively-driven differentiation of sex-related chromosomes may be rare in such inbred organisms. As an alternative to selective explanations, neutral inversions may accumulate near sex-determining regions (Ironside 2010; Ponnikas *et al.* 2018), or reduced recombination may occur as a side-consequence of silencing transposable elements that spread in and near sex-determining regions (Kent *et al.* 2017).

Most theories about the evolution of sex chromosomes are based on studies of organisms in which sex is determined at the diploid stage (*e.g.*, animals) (Bergero and Charlesworth 2009; Charlesworth 2016; Wright *et al.* 2016). However, the suppression of recombination in sex-determining regions can also occur in organisms in which sex is determined at the haploid stage, such as algae or bryophytes (Coelho *et al.* 2018). A progressive spread of recombination suppression on sex chromosomes can occur when there is antagonistic selection between the two sexes (Immler and Otto 2015). In principle, this could also apply to fungi, in which mating type is controlled at the haploid stage, provided that there are traits for which optima differ between mating types. However, there may be few adaptive differences between haploid cells of different mating types other than mating-type determination itself and sometimes mitochondrial inheritance (Xu 2005; Billiard *et al.* 2011). Differences in gene expression levels have been found between haploid cells of different mating types in some fungi (Samils *et al.* 2013; Grognet *et al.* 2014; Fontanillas *et al.* 2015). However, such differences in expression levels between fungal mating types may be due to degeneration following recombination suppression, rather than adaptive differences between the mating types (Fontanillas *et al.* 2015).

The anther-smut fungus *Microbotryum lychnidis-dioicae* has mating-type chromosomes with large regions devoid of recombination. The cessation of recombination occurred in several successive steps, with six different evolutionary strata dating from 0.9 to 2.1 million years ago. One of the evolutionary strata, known as the black stratum (Branco *et al.* 2017), evolved to link the two mating-type loci controlling pre- and post-mating compatibility, respectively (Figure 1). Such linkage is beneficial when organisms undergo selfing as their main mode of sexual reproduction, as it maximizes the chances of compatibility among the haploid products of meiosis from a single diploid genotype: only two mating types are produced in a progeny with linked mating-type loci against four mating types with unlinked mating types (Nieuwenhuis *et al.* 2013; Branco *et al.* 2017). The other evolutionary strata, all given names based on colors (Branco *et al.* 2017), occurred in successive steps and link genes not involved in mating-type determination to the mating-type genes (Figure 1). The purple, blue and orange strata pre-date the black stratum, and occurred while the mating-type loci were still located on different chromosomes, at the basis of the *Microbotryum* clade, while the red and green strata are younger than the event linking the two mating type loci through the black stratum (Branco *et al.* 2017). A similar stepwise progression of recombination suppression along mating-type chromosomes also occurred independently in other *Microbotryum* fungi, trapping different gene sets (Branco *et al.* 2018). Only small recombining regions remained at both ends of mating-type chromosomes, called pseudo-autosomal regions (PARs).

**Figure 1:**
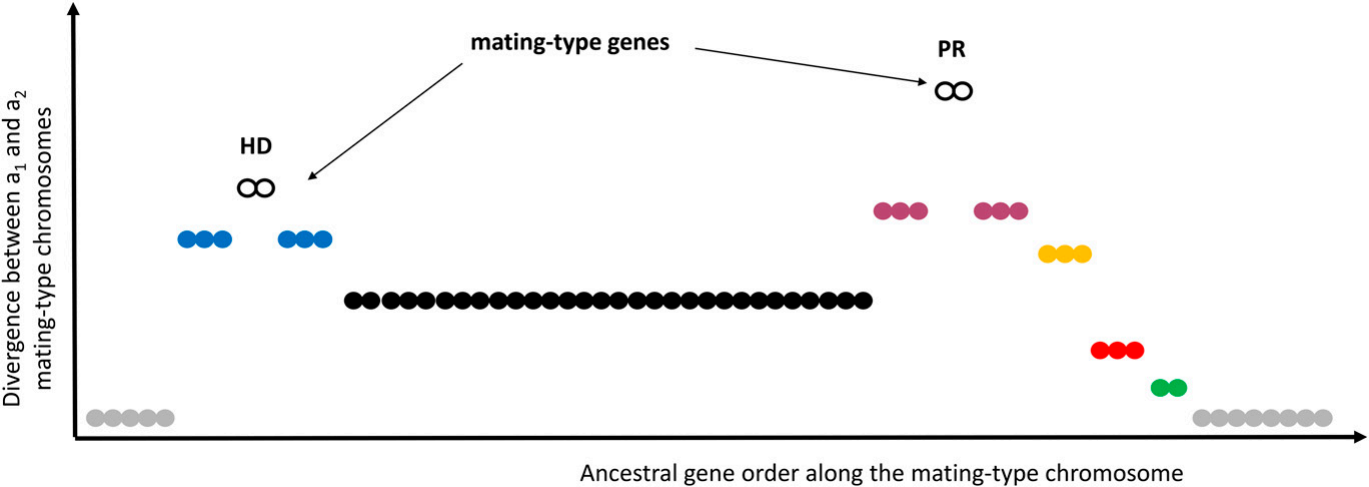
Schematic representation of the evolutionary strata on the mating-type chromosome of *Microbotryum lychnidis-dioicae*. The per-gene synonymous divergence between alleles (y-axis) represents relative timing of the suppression of recombination steps plotted along the ancestral gene order (x-axis). The PR and HD gene clusters (open black circles) show the most ancient divergences. They control pre- and post-mating compatibility, respectively, and encompass several ancestrally linked mating-type genes. The sequence of suppression of recombination begins around each of the mating-type loci, generating the blue and purple evolutionary strata. Recombination suppression then spread distally to the PR locus, creating the orange stratum. The event that linked the two mating-type loci and their surrounding strata generated the black stratum. The suppression of recombination then spread further outwards distal to the PR locus, creating the red and then the green strata. The pseudo-autosomal regions, which are still recombining, are shown in gray. Only the evolutionary strata shown in black (open or closed black circles) involve linking mating-type genes.

These fungi have no ‘female’ or ‘male’ functions (displaying isogamous gametes), and sexual antagonism should not, therefore, have driven the development of these evolutionary strata. In addition, the selfing mating system in these fungi may render challenging to maintain sexually antagonistic variation, if any (Gregorius 1982; Charlesworth and Wall 1999; Jordan and Connallon 2014). Furthermore, the brevity of the haploid stage suggests that there would be little opportunity for ploidally antagonistic selection (Immler and Otto 2015).

Antagonistic selection could instead act between haploid mating types, but there is currently little evidence for such antagonism in *Microbotryum* fungi. Our understanding of the life cycle (Figure 2) suggests that the haploid phase is very brief which limits the possibility of mating-type specific antagonistic selection or ploidally antagonistic selection (*i.e.*, differential selection between haploid and dikaryotic phases). Most mating events occur rapidly after meiosis, between products of the same meiotic tetrad, causing high levels of inbreeding (Hood and Antonovics 1998; Hood and Antonovics 2000; Hood and Antonovics 2004; Schäfer *et al.* 2010). Mating generates infectious dikaryotic (n+n) hyphae that penetrate the plant. While haploid sporidia can multiply *in vitro* on media with a high sugar content and in flower nectar (Schäfer *et al.* 2010; Golonka and Vilgalys 2013), it remains unclear whether this stage is of any biological relevance (Hood and Antonovics 2000; Hood and Antonovics 2004; Granberg *et al.* 2008). Dikaryotic hyphae are not thought to invade the plant via flowers but rather by penetration at the junction of the anticlinal epidermis on vegetative tissues with low sugar content (Schäfer *et al.* 2010). It is often the case that spore-bearing anthers are present early in the season in the first flower (Alexander and Maltby 1990; Alexander and Antonovics 1995), implying that infection occurred at the vegetative stage prior to the development of the first flower. Furthermore, male plants develop disease at least as frequently as female plants (Zillig 1921; Alexander 1989; Alexander 1990; Thrall and Jarosz 1994; Alexander and Antonovics 1995; Biere and Antonovics 1996; Biere and Honders 1998), despite the fact that male flowers fall rapidly after pollinator visits (Kaltz and Shykoff 2001), consistent with the view that infection does not occur through floral tissues where haploid cells might grow.

**Figure 2:**
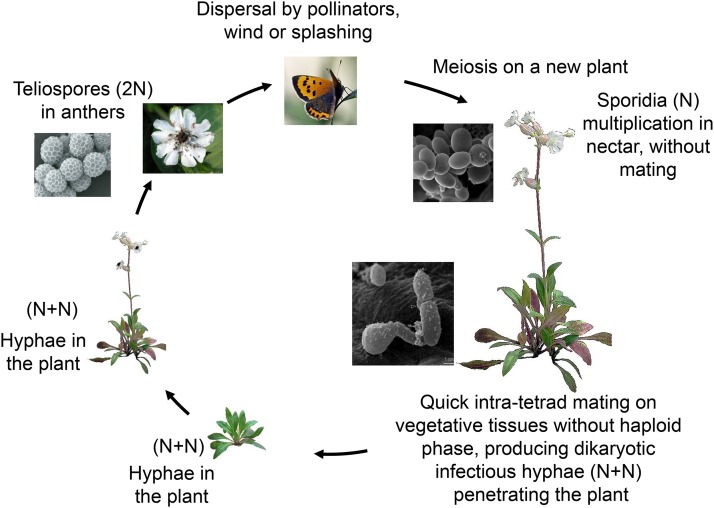
Life cycle of the anther-smut fungus *Microbotryum lychnidis-dioicae*. Diploid (2N) teliospores are produced in anthers of diseased plants, being heterozygous at all mating-type genes. The teliospores are dispersed to healthy plants by pollinators, wind or splashing. Once on a new plant, teliospores undergo meiosis. In the nectar of flowers, haploid sporidia (N) multiply clonally without mating until the flower wilts, after which flowers fall in male plants. On vegetative tissues where teliospores fall from flowers or by splashing, quick intra-tetrad mating occurs, preventing any haploid phase, producing dikaryotic infectious hyphae (N+N) penetrating the plant. In dikaryotic hyphae, there is exactly one nucleus of each mating type in each cell, preventing competition between mating types for replication and transmission. The flowers produced from infected meristems will produced diseased flowers. Pictures from López-Villavicencio *et al.* (2007) and Schäfer *et al.* (2010) © Canadian Science Publishing or its licensors.

Most tellingly, alleles that are lethal in the haploid phase and linked to mating-type loci have been found in up to 50% of *M. lychnidis-dioicae* strains in natural populations (Kaltz and Shykoff 1997; Oudemans *et al.* 1998; Thomas *et al.* 2003). The maintenance of such alleles can only be explained by a lack of the free-living haploid phase in nature and mating within the tetrad. Consistent with this inference, estimates of selfing rates are extremely high in natural populations, at about 95% (Giraud *et al.* 2005; Vercken *et al.* 2010), as shown by the almost complete homozygosity of the autosomes (Vercken *et al.* 2010; Branco *et al.* 2018). The limited nature of a free-living haploid phase in *Microbotryum* fungi raises a challenge to any hypothesis for the successive spread of suppressed recombination on the sex chromosomes that relies on antagonistic selection (given that the mating types are only separate in the haploid phase) or ploidally antagonistic selection.

Even if there is a free-living haploid phase, few traits other than those determined by mating-type genes may make a “better a_1_” but a “worse a_2_”, or vice versa, which would be a prerequisite for recombination suppression by antagonistic selection. Similarly, we hypothesized that there would be little evidence for alleles favored in one of the mating types but disfavored in the dikaryotic phase, a prerequisite for recombination suppression by ploidally antagonistic selection (Immler and Otto 2015). Nevertheless, even transient selection acting on a few loci subject to antagonistic selection involving the haploid phase could drive the spread of suppressed recombination on the mating-type chromosomes. For example, inheritance of mitochondria may be asymmetric between mating types in *M. lychnidis-dioicae*, although not completely uniparental (Wilch *et al.* 1992) where the fungus was still named *Ustilago violacea*), potentially introducing selection in the haploid phase. We thus examined footprints of selection in *M. lychnidis-dioicae* for signs of antagonistic selection between the mating types or between the haploid and the dikaryotic phase, focusing on genes belonging to the evolutionary strata that are not involved in mating-type locus linkage, known as the “color strata” (Branco *et al.* 2017) (Figure 1). For the “black” stratum that evolved for linking mating-type loci (Figure 1), no further evolutionary explanations are required. Genes under antagonistic selection between mating types would, by definition, have important and different roles in the haploid a_1_ or a_2_ mating-type cells. Such genes could therefore be expressed at higher levels in the haploid stage (in at least one mating type) than in the dikaryotic stage (heterozygous for mating type). It is indeed common that genes important in a particular life stage are upregulated in that stage, especially in fungi, in which, for example, genes involved in pathogenicity are often upregulated in the host plant (Seitner *et al.* 2018; Zhang *et al.* 2018). If genes undergoing antagonistic selection drive stratum evolution beyond mating-type genes, these genes should be present in the “color strata”. We thus sought to determine whether genes are more often haploid-upregulated in color strata compared to autosomes (contrasting expression in haploid a_1_ and/or a_2_ cells *vs.* in dikaryons), although we recognize that a single gene with antagonistic selection may be sufficient to drive recombination cessation in each stratum, which would be difficult to identify. This rationale is similar to using an enrichment in sex-biased genes in young evolutionary strata as evidence for sexually antagonistic selection driving sex chromosome evolution (Charlesworth 2017). Antagonistic selection between mating types may also drive differences in gene expression patterns between the two mating types, allowing the opposing selection pressures to be partially or fully resolved. We thus asked whether genes upregulated in the haploid phase also displayed significant differential expression between mating types more often in color strata than in autosomes, indicating that such strata have witnessed more antagonistic selection in the past.

Genes under antagonistic selection may alternatively show signs of divergent selection between mating types in terms of protein sequence. We tested whether the color evolutionary strata were enriched in genes with signs of divergent selection by looking at the ratio of non-synonymous *vs.* synonymous substitutions. Two main issues in such enrichment tests have to be kept in mind, however: (i) finding no enrichment in genes under differential expression or divergent selection does not constitute definitive evidence for the lack of antagonistic selection; (ii) finding enrichment in genes under differential expression or divergent selection does not constitute definitive evidence for antagonistic selection; (iii) divergent selection in expression or sequence may occur after recombination suppression. More generally, identifying loci under antagonistic selection is notoriously difficult even under ideal scenarios. Nevertheless, such tests contribute to our global understanding of selective pressures occurring in evolutionary strata.

We used published expression data to address these questions (Fontanillas *et al.* 2015; Perlin *et al.* 2015), together with stratum delimitation based on high-quality genome data (Branco *et al.* 2017). Gene expression in various stages in *M. lychnidis-dioicae* (Perlin *et al.* 2015) and in haploid cells of different mating types (Fontanillas *et al.* 2015) were produced in previous studies. The life stages investigated were: (i) haploid yeast forms of separate mating types grown on water agar, under conditions inducing mating *in vitro* when mating types are mixed (Hood and Antonovics 2004) and, thus, simulating the stage at which separate mating types are present on the meristem before mating and plant infection; (ii) haploid yeast forms of separate mating types grown in medium with a high glucose content where they multiply by mitosis; these conditions simulate the stage at which haploid sporidia undergo mitosis in flowers; (iii) the dikaryotic parasitic stage, called “n+n” for dikaryotic stage with two unfused nuclei per cell, before karyogamy, studied in infected plants (Perlin *et al.* 2015). We used these gene expression data, as well as sequence data, to test whether antagonistic selection between mating types played a role in the spread of recombination suppression beyond coupling the mating-type genes in *M. lychnidis-dioicae*.

## Materials and Methods

### Datasets

We used published gene expression data to test for antagonistic selection in *M. lychnidis-dioicae* (Fontanillas *et al.* 2015; Perlin *et al.* 2015): Suppl. Table S1 https://trace.ncbi.nlm.nih.gov/Traces/study/?acc=+PRJNA246470&go=go).

We also used published gene predictions, assignations to genomic compartments and orthologous group reconstruction (Branco *et al.* 2017; Branco *et al.* 2018).

### Differential expression analyses

We performed differential expression analyses to identify genes evolving under antagonistic selection between mating types, as we expected such genes to be (i) upregulated in the haploid phase compared to the dikaryotic phase and/or (ii) upregulated in one mating-type compared to the opposite mating-type. We performed a pseudo-alignment of each read set from the published RNAseq experiments (Suppl. Table S1) against each of the predicted set of coding-sequences from the a_1_ and a_2_ haploid genomes of the Lamole *M. lychnidis-dioicae* strain (Badouin *et al.* 2015) (Suppl. Table S2), using algorithms implemented in Kallisto v. 0.45.0 (Bray *et al.* 2016). Kallisto performs an RNAseq read count quantification through a k-mer and De Bruijn graph approach, allowing ultra-fast quantification and calculation of standard errors using bootstraps. We ran Kallisto with 100 bootstraps samples and a sequence-based bias correction.

We then used the pseudo-alignment outputs to perform differential expression analyses using the Sleuth R package (Pimentel *et al.* 2017). The statistical methods implemented in Sleuth allow accurate estimation of differential expression levels and of their significance using the entire quantification variance from the bootstraps. To estimate the significance of the differential expression levels, we used the likelihood-ratio test (LRT) implemented in Sleuth to compare linear models assuming different parameters to explain the variance from the abundance estimates for each sample. Specifically, we compared a model assuming the variance to be explained only by biological replicates to models that take into account the replicates and either the life stage (*i.e.*, haploid or dikaryotic) or the mating-type (either a_1_ or a_2_). After the LRT, the Sleuth package returns a *q-value* per coding-sequence, *i.e.*, the corrected *p-value* following the Benjamini-Hochberg correction for reducing the false discovery rate (FDR) due to multiple testing; we chose a threshold of 0.001 for the *q-value* (corresponding to a FDR of 0.001), below which we considered the differential expression to be significant (analyses with higher thresholds did not change the global patterns and appeared not stringent enough, with most genes differentially expressed, Figure S1). We performed analyses of upregulation in the haploid stage using the set of coding-sequences from either the a_1_ or a_2_ haploid genome of the Lamole *M. lychnidis-dioicae* strain as reference. We only present the analyses based on the a_1_ reference as results based on the a_2_ reference yielded the same patterns and conclusions. The Suppl. Tables S1 and S2 present the accession numbers of the data used as well as pseudo-alignment statistics.

We investigated whether the non-recombining regions, and, more specifically, the color strata not involving mating-type genes, contained a higher proportion of genes upregulated in the haploid phase than autosomes, by performing chi-squared tests. We thus tested whether the genes more strongly expressed in at least one haploid condition/mating type than at the dikaryotic stage were more frequent on the mating-type chromosome than on autosomes. We first performed a global chi-squared test comparing the various genomic compartments (autosomes, PARs, black and color strata). We then compared the proportion of genes upregulated in the haploid phase in the color strata and the autosomes. As the question of the evolutionary origin of the strata not involving mating-type genes was the same for all the color strata and there were few genes in the color strata (Table 1), we pooled the genes from all color strata for the various tests to improve power.

**Table 1:** Counts of genes in *Microbotryum lychnidis-dioicae* with expression data that could be assigned to the various chromosomal locations and those with differential expression (threshold 0.001) between life stages, respectively for the autosomes, pseudoautosomal regions (PARs) of the mating-type chromosome, and non-recombining region (NRR) of the mating-type chromosome, separated into the different evolutionary strata (blue, purple, black, orange, red and green)

	Number of assigned genes	Number of genes upregulated in at least one haploid stage/mating type	Percentage of genes upregulated in at least one haploid stage/mating type
Total	9983	1534	15.37%
Autosomes	9083	1454	16.01%
PAR	137	13	9.49%
NRR black stratum	698	52	7.45%
NRR color strata (sum)	65	15	23.08%
Blue stratum	21	4	19.05%
Green stratum	3	1	33.33%
Orange stratum	7	0	0.00%
Purple stratum	9	4	44.44%
Red stratum	25	6	24.00%

For the genes upregulated in the haploid compared to the dikaryotic phase, we then investigated whether the differential expression levels were greater in color strata than on the autosomes. We hypothesized that, if the genes on the color strata had particularly important roles in the haploid phase, the haploid upregulation relative to the dikaryon might be stronger in the color strata than in autosomes. We performed pairwise Wilcoxon signed-rank tests to compare the distribution of the differential expression level of genes upregulated in the haploid phase between autosomes, the black stratum and the pooled color strata, for each mating type and each medium. The Sleuth package returns the differential expression level as a “beta” value. In order to get a differential expression level index similar to the classically used log2 fold-change, we used the option “transform_fun_counts = function(x) log2(x + 0.5)” in the data preparation steps, as advocated in the Kallisto and Sleuth methods (sleuth_prep() R function; https://github.com/pachterlab/sleuth/issues/59).

We also investigated whether the genes upregulated in the haploid phase displayed differential expression between the a_1_ and a_2_ mating types, and whether the difference level, if any, was greater than for other genes. We only compared genes from the same genomic compartment (black stratum or pooled color strata) in order to compare genes with similar levels of degeneration due to recombination suppression, as degeneration can lead to differential expression between mating types without selection for it (Fontanillas *et al.* 2015). We used the differential expression levels as given by the beta values between the a_1_ and a_2_ mating types for genes upregulated in the haploid phase. We compared the distributions of differential expression level of genes upregulated during the haploid phase with those of all other pooled differentially expressed genes in the same genomic compartment, in a Wilcoxon signed-rank test in R. We also identified the genes significantly upregulated in one mating type compared to the other, either in water or rich medium. We also investigated genes differentially expressed between mating types in haploid phases at the 0.001 threshold.

### Positive selection tests

If antagonistic selection drove the spread of recombination suppression beyond mating-type genes, generating the color evolutionary strata, then we could alternatively expect the genes involved in functions specific to alternative mating types to show specialization in the a_1_ or a_2_ mating type, with alleles encoding proteins with different sequences. We therefore tested whether the genes present in color strata displayed footprints of divergent selection between the alleles associated with the alternative mating types, in the form of significantly higher non-synonymous divergence (dN) than would be expected from the synonymous (neutral) substitution rate (dS). Ratios of dN/dS, known as ω, and the significance of positive selection assessed by comparing the likelihood of different sequence evolution models, were inferred with CODEML in the PAML package (Yang 2007). For all genes for which both a_1_ and a_2_ alleles were present, in the mating-type chromosome and in autosomes as control, we performed branch-site tests of positive selection (“Test 2” in the PAML manual, 2017 version). For each gene, we tested whether a model of evolution allowing sites to evolve with a different ω, and possibly >1, in the branch of the alleles associated with the a_1_ or a_2_ mating type in *M. lychnidis-dioicae* was more likely than a model with ω ≤ 1 in all branches and sites and with similar values in all branches. For modeling sequence evolution and the direction of nucleotide changes, we used, as background branches, the sequences of genes in the a_1_ and a_2_ mating types in *M. lagerheimii* and *M. saponariae* (with recombining mating-type chromosomes), and, as the focal (foreground) branch, either the a_1_ or a_2_ sequence of *M. lychnidis-dioicae*. The input tree was the focal gene tree, as recombination suppression and gene conversion lead to gene-specific genealogies with more or less *trans*-specific polymorphism for a_1_ and a_2_ alleles (Branco *et al.* 2017; Branco *et al.* 2018). To build the gene trees, we used RAxML (Stamatakis 2006) under a GTRGAMMA model, with the orthologous sequences from both mating types in each of the three species *M. lychnidis-dioicae*, *M. lagerheimii* and *M. saponariae*, aligned using the codon-based approach implemented in translatorX (Abascal *et al.* 2010). In the first model (model A, specified in the PAML control file as: model = 2, NSsites = 2, omega = 0, fix_omega = 0.2), different classes of site were allowed: class 0 with 0 < ω < 1 in the background and foreground branches, class 2a with 0 < ω < 1 in the background branch and ω > 1 in the foreground branch, class 2b with ω =1 in the background branch and ω > 1 in the foreground branch. In the null model without positive selection (null model A, specified in the PAML control file as: model = 2, NSsites = 2, omega = 1, fix_omega = 1), three classes of sites were allowed, with either ω < 1 or ω = 1 in both the background and foreground branches, or ω < 1 in the background branches and ω = 1 in the foreground branch. Likelihood ratio tests (LRTs) were performed to compare the two models, with one degree of freedom, as suggested in the PAML documentation, and ω values were inferred for the different classes of sites. Because low levels of synonymous divergence can artificially inflate ω estimates, which would then be unreliable, we discarded the likelihood values resulting from the A model when the ω estimated of the foreground branch was higher than five in either the 2a or 2b site class, as typically recommended (Pond and Muse 2005; Chamary *et al.* 2006; Stoletzki and Eyre-Walker 2011). Such biases can be particularly problematic in non-recombining regions where non-synonymous substitutions may accumulate due to relaxed selection. We nevertheless also present results without filtering.

In addition, we plotted the per-gene d_N_/d_S_ between the a_1_ and a_2_-associated alleles along the *M. lagerheimii* ancestral-like gene order of the mating-type chromosome, using the d_N_ and d_S_ values computed in the yn00 program (Yang and Nielsen 2000; Yang 2007).

### Data availability

This manuscript uses previously published data, available at https://trace.ncbi.nlm.nih.gov/Traces/study/?acc=+PRJNA246470&go=go.

The two Supplementary Figures and the seven Supplementary Tables are available at FigShare: https://doi.org/10.25387/g3.8024813.

## Results

### Upregulation in the haploid stage and/or in one mating type

**Figure 3:**
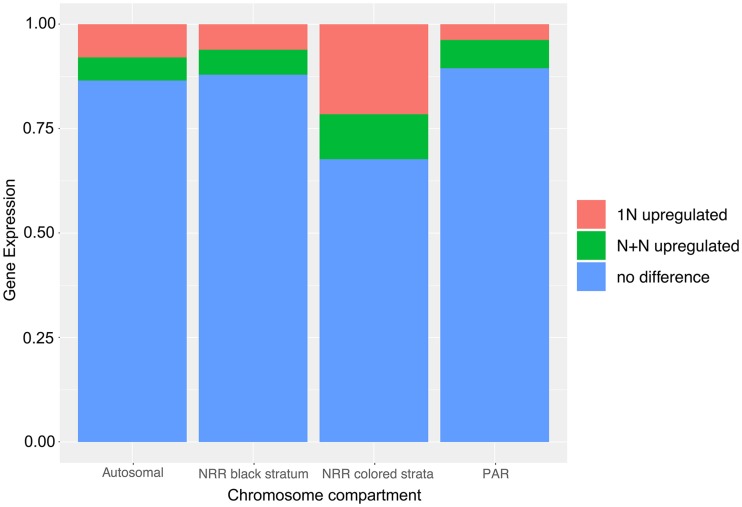
Differential expression in *Microbotryum lychnidis-dioicae*. Proportions of genes upregulated in at least one haploid stage (in red, 1N upregulated), upregulated at the dikaryotic stage (in green, N+N upregulated) or showing no differential expression (in blue). Expression level was considered significantly different at the 0.001 threshold. Different thresholds for significance did not change the patterns notably (Suppl. Figure S1). Genes are separated according to their genomic compartment: autosomes, pseudoautosomal regions (PARs) of the mating-type chromosome, non-recombining region (NRR) of the mating-type chromosome, and into the black *vs.* color evolutionary strata.

Among the 12,254 predicted genes in *M. lychnidis-dioicae*, we were able to assign 9,983 genes studied for expression to chromosomal locations, *i.e.*, on autosomes, pseudo-autosomal regions (PARs) of the mating-type chromosome, the ‘black’ stratum of the non-recombining region (NRR) linking the two mating-type loci, and the various evolutionary “color strata” of the non-recombining regions not involving mating-type genes (the ‘blue’, ‘green’, ‘orange’, ‘red’ and ‘purple’ strata, Figure 1). Genes differentially expressed (*q-value* ≤ 0.001) between at least two life stages represented 15% of the assigned genes and were distributed among the various genome compartments (Figure 3 and Table 1). Most (95%) of the genes upregulated in the haploid phase nevertheless resided on autosomes (Table 1).

**Figure 4:**
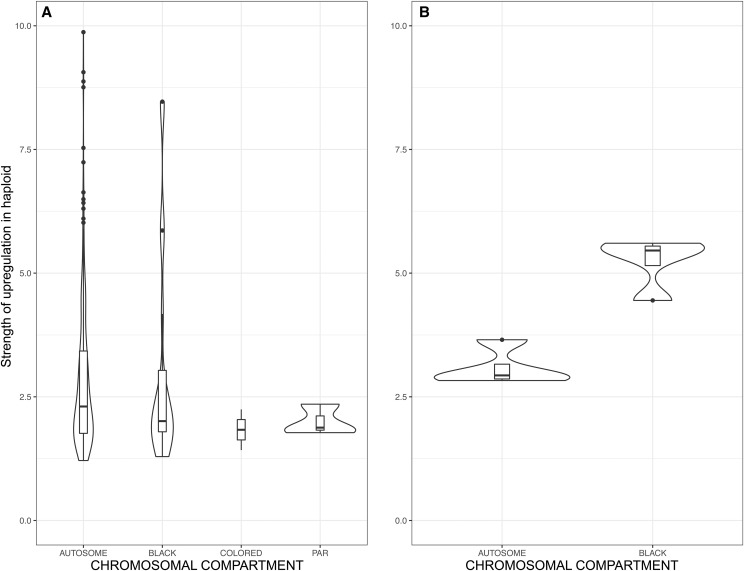
Strength of haploid upregulation in the different genomic compartments in *Microbotryum lychnidis-dioicae*. Violin plots of the differential expression levels (beta values, calculated in a similar way as the usual log_2_ fold-change) of the haploid compared to the dikaryotic phase for the various genomic compartments (autosomes, black stratum, color strata and PARs), with haploids grown in: A ‘rich’ or B ‘water’ media (dikaryotic expression was measured *in planta*). In panel B, no genes were found upregulated in water in color strata or PARs.

Significant differences were detected in the proportion of genes upregulated in a haploid phase compared to the dikaryotic phase among the various genomic compartments, *i.e.*, autosomes, PARs, black and color strata (Figure 3; chi-squared = 43.122, df = 3, *p*-value = < 2.319e-09). The significance was however mainly driven by the black stratum and the PARs being depleted in genes upregulated in the haploid phase (Figure 3 and Table 1). The color strata displayed no significant enrichment relative to autosomes in genes upregulated in the haploid phase (chi-squared = 2.3925, df = 1, *p*-value = 0.1219). Only 15 genes were found to be upregulated in the haploid phase and residing in the color strata (Table 1), and they all were upregulated in the rich medium, none in the water medium (Figure 4). Using higher thresholds for significant differences in expression levels did not change the patterns notably regarding the relative proportions of genes upregulated in a haploid stage among genomic compartments and the high proportions of genes differentially expressed between life history stages suggested that these thresholds were not stringent enough (Suppl. Figure S1). The putative functions of the genes upregulated in at least one haploid stage/mating type in the black or color strata did not correspond to functions reasonably expected to be advantageous in one mating type but not the other, as could be functions related to mitochondria inheritance (Suppl. Table S3). These findings altogether provide little support that antagonistic selection was a major driver of the spread of recombination suppression beyond mating-type genes.

We then tested whether the genes identified above as upregulated in the haploid cells had particularly large differences in expression between the dikaryotic phase and the haploid phases, comparing haploid expression, in either rich or water media, to dikaryotic expression *in planta*. The differential expression level for haploid-upregulated genes was not stronger for genes in the color strata than for genes in autosomes (Suppl. Table S4; Figure 4). Genes in the color strata even showed less variation and fewer extreme values in differential expression (Figure 4). Haploid upregulation was stronger in the black stratum than in autosomes in the water medium, although the test was not significant anymore when applying a Bonferroni correction for multiple testing (Figure 4B; Suppl. Table S4).

**Figure 5:**
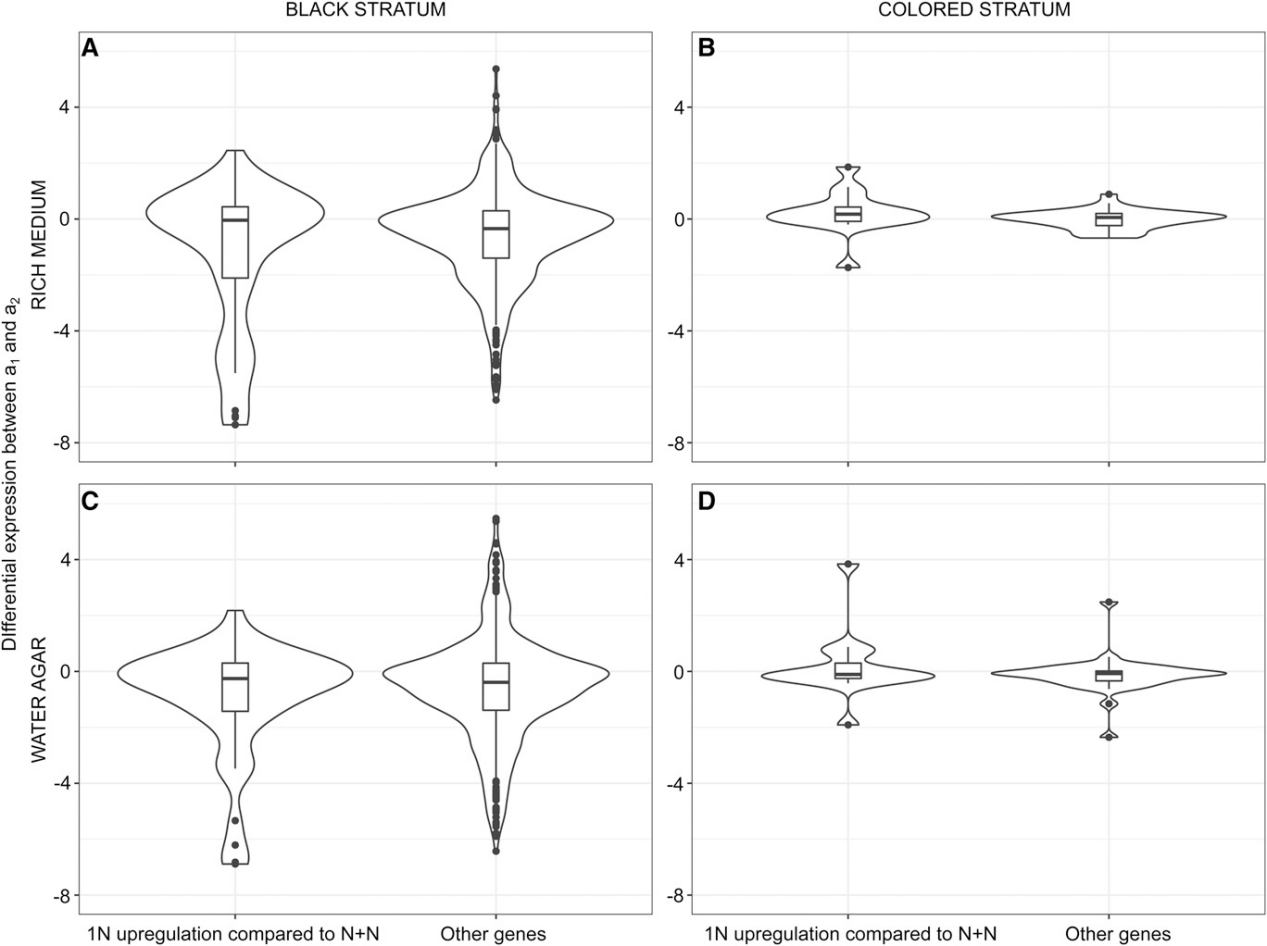
Strength of differential expression between mating-types in *Microbotryum lychnidis-dioicae*. Violin plot of differential expression (beta values, calculated in a similar way as the usual log_2_ fold-change) between the a_1_ and a_2_ mating types at haploid stages in *Microbotryum lychnidis-dioicae*, for genes found upregulated in at least one haploid stage compared to the dikaryotic stage and for the other genes (either upregulated in the dikaryon or without differential expression), in the black stratum (A and C) or the color strata (B and D), on rich medium (A and B) or water (C and D).

We found that the genes in the color or black strata that were upregulated in the haploid compared to the dikaryotic phase displayed no greater difference either in expression level between the a_1_ and a_2_ mating types than other genes (Figure 5; Suppl. Table S5), which is again not consistent with the antagonistic selection hypothesis. The color strata outlier (MvSl-1064-A1-R4_A1g01162) that showed higher haploid differential expression between a_1_ and a_2_ in both water agar and rich medium belonged to the blue stratum, one of the oldest strata. This gene had no putative function, and a BLASTp search did not yield any further insight into its function.

There were only eight genes in the genome with significant differential expression between mating types, all in the water medium, none residing in color strata. The genes with significant differential expression between mating types included the pheromone receptor gene itself, two genes in autosomes and five in the black stratum, with no obvious function that can be related to antagonistic selection (Suppl. Table S6; the enrichment in the black stratum relative to autosomes was not significant; Fisher exact test, *P* = 0.08) The finding of a lack of genes with differential expression between mating types in color strata again does not support the hypothesis that mating-type antagonistic selection would drive evolutionary strata of recombination suppression.

### Divergent selection between mating types

We then investigated whether the color strata were enriched in genes with signs of divergent selection between mating types, with amino-acid substitutions more frequent between the a_1_ and a_2_ mating types than would be expected on the basis of neutral (synonymous) substitution rates (*i.e.*, ω = dN/dS >1). For each gene, we considered, as background branches, the sequences of genes in the a_1_ and a_2_ mating types of the outgroup *M. lagerheimii* and *M. saponariae* (species with recombining mating-type chromosomes), and as the focal (foreground) branch, either the a_1_ or a_2_ sequence of *M. lychnidis-dioicae* (Suppl. Figure S2). We determined whether models allowing sites with ω >1 in the foreground branches were more likely (Suppl. Figure S2). For comparison, we also ran the analyses on autosomal genes, PAR genes, and genes in the black stratum.

Likelihood ratio tests, performed after filtering to remove low d_S_ values that may generate unreliable d_N_/d_S_ estimates (Pond and Muse 2005; Chamary *et al.* 2006; Stoletzki and Eyre-Walker 2011), indicated that the model with divergent selection between mating types was significantly more likely than the alternative in very few genes in non-recombining regions (Table 2): only two genes in the black stratum were found to evolve under positive selection, one in each of the a_1_ and a_2_ mating-type chromosome, and none in the color strata. The color strata were thus not enriched in genes under positive selection compared to the autosomes, they even seemed depleted in genes under diversifying selection between mating types. The putative functions of the genes under positive selection did not appear likely involved in antagonistic selection between mating types (Suppl. Table S3).

**Table 2:** Numbers and proportions of genes for which the null model or the model with selection was the most likely in PAML’s branch-site tests of positive selection for each gene in autosomes, in PARs, in the black and color evolutionary strata, and for genes upregulated at the haploid stage

a_1_ mating-type	Null model	Model with selection	Model with selection among upregulated
Autosomal genes (MC02)	6286	28	3
PARs	89	0	0
Black stratum	118	1	0
Purple stratum	8	0	0
Blue stratum	18	0	0
Orange stratum	7	0	0
Red stratum	23	0	0
Green stratum	3	0	0
Color strata (pooled)	59	0	0
**a_2_ mating-type**			
Autosomal genes (MC02)	6262	41	7
PARs	89	0	0
Black stratum	105	1	0
Purple stratum	8	0	0
Blue stratum	17	0	0
Orange stratum	5	0	0
Red stratum	24	0	0
Green stratum	3	0	0
Color strata (pooled)	57	0	0

Without filtering for low d_S_ values, there were 11 genes with signs of positive selection in one of the mating types in color strata (four in the blue stratum, three in the red stratum, two in the orange stratum and two in the purple stratum; Suppl. Table S7). Only one of these 11 genes, located in the purple stratum, was significantly upregulated in the haploid phase (Suppl. Table S7). There were 47 genes with signs of positive selection in the black stratum, three being haploid upregulated, and 140 in autosomes, 17 being haploid upregulated. There was a significant enrichment in the both the black and color strata compared to autosomes for genes with signs of positive selection (Fisher tests, *P* = 2.2 ×10^−16^ for the black stratum, *P* = 1.36×10^−8^ for the color strata). The enrichment in genes with both signs of positive selection and haploid upregulation was significant only for the black stratum and not for the color strata (Fisher tests, d.f.=1, *P* = 0.0025 for the black stratum, *P* = 0.1218 for the color strata). The putative functions of the genes with signs of positive selection (Suppl. Table S7) did not suggest any role for antagonistic selection, except perhaps a function linked to mitochondria stability in the black stratum (MvSl-1064-A1-R4_A1g00541, see discussion).

**Figure 6:**
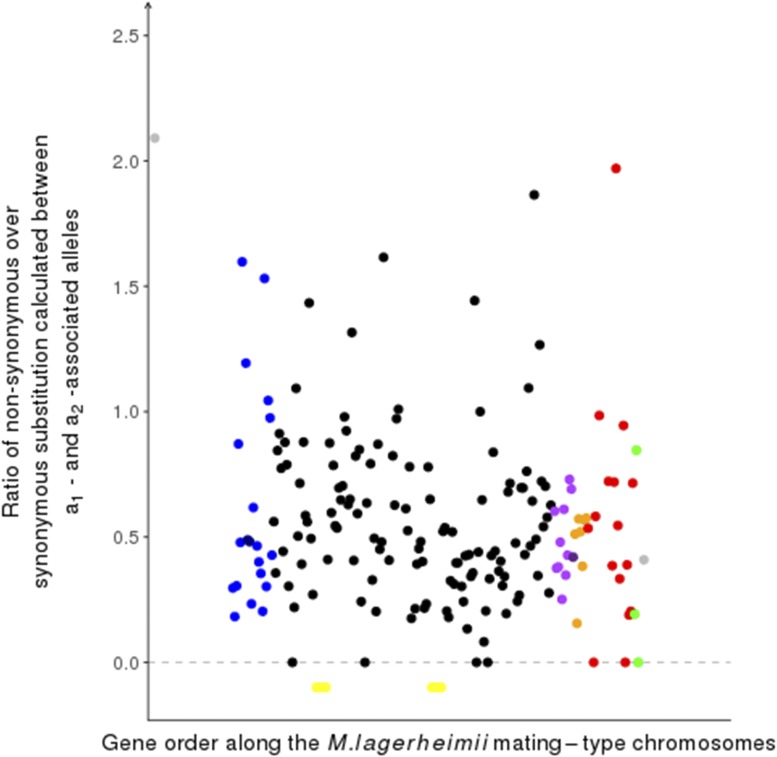
Per-gene non-synonymous over synonymous (d_N_/d_S_) differences between a_1_-a_2_ associated mating types along the mating-type chromosomes in *Microbotryum lychnidis-dioicae*. Genes are located according to the ancestral-like gene order (*i.e.*, gene order from *M. lagerheimii*) and evolutionary strata are indicated by their colors. Ancestral location of centromeres (before chromosomal fusion) are indicated in yellow; d_N_/d_S_ values could not be computed for most of the genes in pseudo-autosomal regions (in gray), as most had null d_S_ values.

The d_N_/d_S_ values between alleles associated with a_1_ *vs.* a_2_ mating types were not higher in the color evolutionary strata (Figure 6). Altogether these findings provide little support for the notion that the specialization of genes with important haploid roles to a_1_ or a_2_ mating types is the predominant force driving recombination suppression in the various color strata.

## Discussion

Our findings that the color strata of the non-recombining mating-type chromosomes were not enriched in genes upregulated in the haploid phase and carried no gene differentially expressed between mating types or under divergent selection after filtering provide little support for the hypothesis that the spread of recombination suppression beyond mating-type genes in *M. lychnidis-dioicae* was due to antagonistic selection between mating types.

Relaxing the filtering of d_N_/d_S_ for high values, we found 11 genes with significant signs of positive selection in color strata. However, the finding that a single one was upregulated in the haploid phase (where mating types are expressed) and their putative functions provided little evidence for antagonistic selection. The single haploid upregulated gene with significant positive selection without filtering among color strata was in the purple stratum and appeared involved in histone deposition, which could be related to recombination suppression (MvSl-1064-A1-R4_A1g00230); this is relevant for the evolution of mating-type chromosomes but unlikely to involve antagonistic selection. Note that a very high d_N_/d_S_ ratio most often represents a biased estimate (when d_S_ is very low, d_N_/d_S_ estimates are not reliable) and filtering them out is recommended and typically done (Pond and Muse 2005; Chamary *et al.* 2006; Stoletzki and Eyre-Walker 2011; Villanueva-Cañas *et al.* 2013). This issue is likely particularly problematic in the highly selfing *M. lychnidis-dioicae*, in which differentiation is very low between a_1_ and a_2_ genomes in a given diploid individual in young evolutionary strata, and in which non-synonymous substitutions accumulate in non-recombining regions due to relaxed selection (Fontanillas *et al.* 2015). In addition, signs of positive selection in evolutionary strata may be due to dominant beneficial mutations that have appeared after recombination suppression and cannot spread to the alternative allele due to lack of recombination and may thus not correspond to divergent selection between mating types. Most of the putative functions of genes with significant signs of positive selection without filtering in fact did not correspond to roles that can be imagined to be under antagonistic selection between mating types. The only function that could be related to antagonistic selection in non-recombining regions was involved in mitochondria stability (MvSl-1064-A1-R4_A1g00541). Such a function may be involved in mitochondria inheritance, which is asymmetric between mating types in *M. lychnidis-dioicae*. Mating type a_2_ progeny inherit ca 90% of a_2_ parental mitochondria, while equal proportions of parental mitochondria are inherited by a_1_ progeny (Wilch *et al.* 1992). This gene was however located in the black stratum and therefore cannot explain the evolution of recombination suppression in the color strata flanking the mating-type loci. Another interesting function among genes with significant signs of positive selection without filtering in the black stratum appeared related to the negative regulation of mitosis (MvSl-1064-A1-R4_A1g00541), which may be important in the mating stage, but for both mating types. In the future, when more high-quality genome assemblies will be available across the *Microbotryum* genus, it may be worth testing for positive selection including more species to estimate dN/dS along shorter branches just before the evolution of the various color strata.

Genes upregulated in the haploid phase (likely to have important roles in this stage where cells are of distinct mating types) similarly appeared unlikely to have evolved under antagonistic selection as they seem to have similar roles in the a_1_ and a_2_ mating types, particularly for the ones located in the color strata. Genes upregulated in the haploid phase did not differ markedly between the a_1_ and a_2_ mating types, in terms of protein sequence or gene expression level. These findings, together with the location of most (95%) of the genes upregulated in the haploid phase on autosomes, support the view that the functions important in the haploid phase are similar between a_1_ and a_2_ cells, which does not meet the expectations of antagonistic selection acting between mating types. The genes of importance at the haploid stage for roles other than mating-type determinism, such as genes involved in haploid mitotic division or the functional process of mating, are likely to perform the same function in both mating types and would therefore not be expected to be selected for linkage to mating-type genes. The putative functions assigned to the genes upregulated in the haploid phase were in fact all general in nature, with no obvious reason for selection for different aspects in the two alternative mating types.

While antagonistic selection is an attractive and theoretically plausible hypothesis for explaining the spread of recombination suppression on sex chromosomes, decades of research have uncovered limited evidence in support of this theory (Beukeboom and Perrin 2014; Wright *et al.* 2016). Alternative hypotheses have been put forward (Ironside 2010; Ponnikas *et al.* 2018), including the successive linkage of genes accumulating deleterious recessive mutations in the margins of the non-recombining region, due to linkage disequilibrium with sex-determining loci. Complete linkage fixes heterozygosity, thereby permanently sheltering heterozygous deleterious recessive mutations. This process has been hypothesized to play a role in *Microbotryum* fungi (Hood and Antonovics 2000; Antonovics and Abrams 2004; Hood and Antonovics 2004; Johnson *et al.* 2005), as the haploid phase is of limited relevance under natural conditions. Alternatively, chromosomal inversions may arise and fix by drift on one sex chromosome but fail to spread to the other sex chromosome if the inversion completely suppresses recombination with the sex determining locus (Ironside 2010). It has also been suggested that transposable element (TE) accumulation in or near the non-recombining portion of sex chromosomes can suppress recombination further, through genomic silencing of the TEs by DNA methylation and/or chromatin modifications (Kent *et al.* 2017). These hypotheses have been very little studied to date, despite their potentially important roles in the spread of recombination suppression on sex and mating-type chromosomes. The existence of evolutionary strata in a fungus without male and female roles, and the lack of evidence for antagonistic selection between mating types, highlights the need to investigate these alternative hypotheses.

Of course, lack of evidence for widespread antagonistic selection is not evidence that antagonistic selection had no role in stratum evolution, and we had little power to detect antagonistic selection acting on only one or a few sites. In particular, the lack of enrichment in genes upregulated in the haploid phase in the color strata still allows the possibility that a single gene under antagonistic selection, in each stratum, drove recombination suppression. Conversely, finding genes under divergent selection in evolutionary strata does not provide strong evidence that antagonistic selection caused recombination cessation, given that alternative hypotheses for stepwise recombination suppression also predict such footprints of divergent selection. For example, heterozygote advantage in the diploid or dikaryotic phase (potentially applying also to associative overdominance, with different deleterious mutations associated with the two sex chromosomes) can promote recombination cessation and lead to a pattern of divergent selection between alleles associated with alternative mating types (Otto 2014 ; Immler and Otto 2015). Furthermore deleterious allele accumulation after recombination cessation is expected to generate patterns of differential expression between mating types (Fontanillas *et al.* 2015). These issues in testing the role of antagonistic selection in stratum evolution are also reasons why alternative hypotheses to antagonistic selection are worth exploring and disentangling. Investigating genomic patterns of transposable element accumulation and their silencing, as well as deleterious mutation accumulation, at the margin of regions lacking recombination, could allow testing if these mechanisms have promoted the spread of recombination cessation. Silencing-based mechanisms would generate new strata without any discernible chromosomal rearrangement and may lead to a more gradual divergence and loss of recombination than an inversion-based hypothesis, for example. The spread of recombination suppression appears more continuous than discrete in some cases, including in some *Microbotryum* species (Branco *et al.* 2018) and other organisms (Bergero and Charlesworth 2009). In *Microbotryum lychnidis-dioicae*, however, discrete strata have been inferred (Figure 1). The evolutionary forces driving these strata remain to be determined. The work presented here finds little evidence that either mating-type antagonistic selection and/or ploidally antagonistic selection are responsible.

## References

[bib1] Abascal F. Zardoya R. Telford M., 2010 TranslatorX: multiple alignment of nucleotide sequences guided by amino acid translations. Nucleic Acids Res. 38: W7–W13 10.1093/nar/gkq291 20435676 PMC2896173

[bib2] Alexander H. M., 1989 An experimental field-study of anther-smut disease of *Silene alba* caused by *Ustilago violacea*- Genotypic variation and disease incidence. Evolution 43: 835–847. 28564198 10.1111/j.1558-5646.1989.tb05181.x

[bib3] Alexander H. M., 1990 Epidemiology of anther-smut infection of *Silene alba* caused by *Ustilabo violacea* - patterns of spore deposition and disease incidence. J. Ecol. 78: 166–179. 10.2307/2261043

[bib4] Alexander H. M. Antonovics J., 1995 Spread of anther-smut disease *(Ustilago violacea)* and character correlations in a genetically variable population of *Silene alba*. J. Ecol. 83: 783–794. 10.2307/2261415

[bib5] Alexander H. M. Maltby A., 1990 Anther-smut infection of *Silene alba* caused by *Ustilago violacea* - Factors determining fungal reproduction. Oecologia 84: 249–253. 10.1007/BF00318280 28312761

[bib6] Antonovics J. Abrams J. Y., 2004 Intratetrad mating and the evolution of linkage relationships. Evolution 58: 702–709. 10.1111/j.0014-3820.2004.tb00403.x 15154546

[bib7] Badouin H. Hood M. E. Gouzy J. Aguileta G. Siguenza S., 2015 Chaos of rearrangements in the mating-type chromosomes of the anther-smut fungus *Microbotryum lychnidis-dioicae*. Genetics 200: 1275–1284. 10.1534/genetics.115.177709 26044594 PMC4574255

[bib8] Bergero R. Charlesworth D., 2009 The evolution of restricted recombination in sex chromosomes. Trends Ecol. Evol. 24: 94–102. 10.1016/j.tree.2008.09.010 19100654

[bib9] Beukeboom L. Perrin N., 2014, pp. 92–93 in The evolution of sex determination, Oxford University Press, Oxford 10.1093/acprof:oso/9780199657148.001.0001

[bib10] Biere A. Antonovics J., 1996 Sex-specific costs of resistance to the fungal pathogen *Ustilago violacea (Microbotryum violaceum)* in *Silene alba*. Evolution 50: 1098–1110. 28565272 10.1111/j.1558-5646.1996.tb02350.x

[bib11] Biere A. Honders S. C., 1998 Anther smut transmission in *Silene latifolia* and *Silene dioica*: Impact of host traits, disease frequency, and host density. Int. J. Plant Sci. 159: 228–235. 10.1086/297543

[bib12] Billiard S. Lopez-Villavicencio M. Devier B. Hood M. Fairhead C., 2011 Having sex, yes, but with whom? Inferences from fungi on the evolution of anisogamy and mating types. Biol. Rev. Camb. Philos. Soc. 86: 421–442. 10.1111/j.1469-185X.2010.00153.x 21489122

[bib13] Branco S. Badouin H. Rodríguez de la Vega R. Gouzy J. Carpentier F., 2017 Evolutionary strata on young mating-type chromosomes despite lack of sexual antagonism. Proc. Natl. Acad. Sci. USA 114: 7067–7072. 10.1073/pnas.1701658114 28630332 PMC5502610

[bib14] Branco S. Carpentier F. Rodríguez de la Vega R. Badouin H. Snirc A., 2018 Multiple convergent supergene evolution events in mating-type chromosomes. Nat. Commun. 9: 2000 10.1038/s41467-018-04380-9 29784936 PMC5962589

[bib15] Bray N. L. Pimentel H. Melsted P. Pachter L., 2016 Near-optimal probabilistic RNA-seq quantification. Nat. Biotechnol. 34: 525–527. Erratum: 34: 888. 10.1038/nbt.3519 27043002

[bib16] Chamary J. V. Parmley J. L. Hurst L. D., 2006 Hearing silence: non-neutral evolution at synonymous sites in mammals. Nat. Rev. Genet. 7: 98–108. 10.1038/nrg1770 16418745

[bib17] Charlesworth B. Charlesworth D., 1997 Rapid fixation of deleterious alleles can be caused by Muller’s ratchet. Genet. Res. 70: 63–73. 10.1017/S0016672397002899 9369098

[bib18] Charlesworth B. Wall J. D., 1999 Inbreeding, heterozygote advantage and the evolution of neo-X and neo-Y sex chromosomes. Proc. Biol. Sci. 266: 51–56. 10.1098/rspb.1999.0603

[bib19] Charlesworth D., 2016 The status of supergenes in the 21st century: recombination suppression in Batesian mimicry and sex chromosomes and other complex adaptations. Evol. Appl. 9: 74–90. 10.1111/eva.12291 27087840 PMC4780387

[bib20] Charlesworth D., 2017 Evolution of recombination rates between sex chromosomes. Philos. Trans. R. Soc. Lond. B Biol. Sci. 372: 20160456 10.1098/rstb.2016.0456 29109220 PMC5698619

[bib21] Coelho S. M. Gueno J. Lipinska A. P. Cock J. M. Umen J. G., 2018 UV Chromosomes and Haploid Sexual Systems. Trends Plant Sci. 23: 794–807. 10.1016/j.tplants.2018.06.005 30007571 PMC6128410

[bib22] Fontanillas E. Hood M. Badouin H. Petit E. Barbe V., 2015 Degeneration of the non-recombining regions in the mating type chromosomes of the anther smut fungi. Mol. Biol. Evol. 32: 928–943. 10.1093/molbev/msu396 25534033 PMC4379399

[bib23] Giraud T. Jonot O. Shykoff J. A., 2005 Selfing propensity under choice conditions in a parasitic fungus, *Microbotryum violaceum*, and parameters influencing infection success in artificial inoculations. Int. J. Plant Sci. 166: 649–657. 10.1086/430098

[bib24] Golonka A. M. Vilgalys R., 2013 Nectar inhabiting yeasts in Virginian populations of *Silene latifolia* (Caryophyllaceae) and coflowering species. Am. Midl. Nat. 169: 235–258. 10.1674/0003-0031-169.2.235

[bib25] Granberg A. Carlsson-Graner U. Arnqvist P. Giles B. E., 2008 Variation in breeding system traits within and among populations of *Microbotryum violaceum* on *Silene dioica*. Int. J. Plant Sci. 169: 293–303. 10.1086/523964

[bib26] Gregorius H. R., 1982 Selection in plant populations of effectively infinite size. 2. Protectedness of a biallelic polymorphism. J. Theor. Biol. 96: 689–705. 10.1016/0022-5193(82)90237-5

[bib27] Grognet P. Bidard F. Kuchl C. Chan Ho Tong L. Coppin E., 2014 Maintaining two mating types: structure of the mating type locus and its role in heterokaryosis in *Podospora anserina*. Genetics 197: 421–432. 10.1534/genetics.113.159988 24558260 PMC4012498

[bib28] Hood M. E. Antonovics J., 1998 Two-celled promycelia and mating-type segregation in *Ustilago violacea (Microbotryum violaceum)*. Int. J. Plant Sci. 159: 199–205. 10.1086/297539

[bib29] Hood M. E. Antonovics J., 2000 Intratetrad mating, heterozygosity, and the maintenance of deleterious alleles in *Microbotryum violaceum (=Ustilago violacea)*. Heredity 85: 231–241. 10.1046/j.1365-2540.2000.00748.x 11012726

[bib30] Hood M. E. Antonovics J. A., 2004 Mating within the meiotic tetrad and the maintenance of genomic heterozygosity. Genetics 166: 1751–1759. 10.1534/genetics.166.4.1751 15126395 PMC1470809

[bib31] Idnurm A. Hood M. E. Johannesson H. Giraud T., 2015 Contrasted patterns in mating-type chromosomes in fungi: Hotspots *vs.* coldspots of recombination. Fungal Biol. Rev. 29: 220–229. 10.1016/j.fbr.2015.06.001 26688691 PMC4680991

[bib32] Immler S. Otto S. P., 2015 The evolution of sex chromosomes in organisms with separate haploid sexes. Evolution 69: 694–708. 10.1111/evo.12602 25582562

[bib33] Ironside J. E., 2010 No amicable divorce? Challenging the notion that sexual antagonism drives sex chromosome evolution. BioEssays 32: 718–726. 10.1002/bies.200900124 20658710

[bib34] Johnson L. J. Antonovics J. Hood M. E., 2005 The evolution of intratetrad mating rates. Evolution 59: 2525–2532. 10.1111/j.0014-3820.2005.tb00966.x 16526501

[bib35] Jordan C. Y. Connallon T., 2014 Sexually antagonistic polymorphism in simultaneous hermaphrodites. Evolution 68: 3555–3569. 10.1111/evo.12536 25311368 PMC4448763

[bib36] Kaltz O. Shykoff J. A., 1997 Sporidial mating-type ratios of teliospores from natural populations of the anther smut fungus *Microbotryum* (equals *Ustilago*) *violaceum*. Int. J. Plant Sci. 158: 575–584. 10.1086/297470

[bib37] Kaltz O. Shykoff J. A., 2001 Male and female *Silene latifolia* plants differ in per-contact risk of infection by a sexually transmitted disease. J. Ecol. 89: 99–109. 10.1046/j.1365-2745.2001.00527.x

[bib38] Kasimatis K. R. Nelson T. C. Phillips P. C., 2017 Genomic signatures of sexual conflict. J. Hered. 108: 780–790. 10.1093/jhered/esx080 29036624 PMC5892400

[bib39] Kent T. V. Uzunović J. Wright S. I., 2017 Coevolution between transposable elements and recombination. Philos. Trans. R. Soc. Lond. B Biol. Sci. 372. pii: 20160458 10.1098/rstb.2016.0458 PMC5698620 29109221

[bib40] López-Villavicencio M. Jonot O. Coantic A. Hood M. E. Enjalbert J., 2007 Multiple infections by the anther Smut pathogen are frequent and involve related strains. PLoS Pathog. 3: e176 10.1371/journal.ppat.0030176 18020704 PMC2077905

[bib41] Mank J. E., 2017 Population genetics of sexual conflict in the genomic era. Nat. Rev. Genet. 18: 721–730. 10.1038/nrg.2017.83 29062057

[bib42] Marais G. A. B. Nicolas M. Bergero R. Chambrier P. Kejnovsky E., 2008 Evidence for degeneration of the Y chromosome in the dioecious plant *Silene latifolia*. Curr. Biol. 18: 545–549. 10.1016/j.cub.2008.03.023 18394889

[bib43] Nieuwenhuis B. P. S. Billiard S. Vuilleumier S. Petit E. Hood M. E., 2013 Evolution of uni- and bifactorial sexual compatibility systems in fungi. Heredity 111: 445–455. 10.1038/hdy.2013.67 23838688 PMC3833681

[bib44] Otto S. P., 2009 The evolutionary enigma of sex. Am. Nat. 174: S1–S14. 10.1086/599084 19441962

[bib45] Otto, S. P., 2014 Selective maintenance of recombination between the sex chromosomes. 27: 1431–1442. 10.1111/jeb.12324 24529284

[bib46] Oudemans P. V. Alexander H. M. Antonovics J. Altizer S. Thrall P. H., 1998 The distribution of mating-type bias in natural populations of the anther-smut *Ustilago violacea* on *Silene alba* in Virginia. Mycologia 90: 372–381. 10.1080/00275514.1998.12026921

[bib47] Perlin M. Amselem J. Fontanillas E. Toh S. Chen Z., 2015 Sex and parasites: Genomic and transcriptomic analysis of *Microbotryum lychnidis-dioicae*, the biotrophic and plant-castrating anther smut fungus. BMC Genomics 16: 461 10.1186/s12864-015-1660-8 26076695 PMC4469406

[bib48] Pimentel H. Bray N. L. Puente S. Melsted P. Pachter L., 2017 Differential analysis of RNA-seq incorporating quantification uncertainty. Nat. Methods 14: 687–690. 10.1038/nmeth.4324 28581496

[bib49] Pond S. K. Muse S. V., 2005 Site-to-site variation of synonymous substitution rates. Mol. Biol. Evol. 22: 2375–2385. 10.1093/molbev/msi232 16107593

[bib50] Ponnikas S. Sigeman H. Abbott J. K. Hansson B., 2018 Why Do Sex Chromosomes Stop Recombining? Trends Genet. 34: 492–503. 10.1016/j.tig.2018.04.001 29716744

[bib51] Samils N. Gioti A. Karlsson M. Sun Y. Kasuga T., 2013 Sex-linked transcriptional divergence in the hermaphrodite fungus *Neurospora tetrasperma* Proceedings of the Royal Society B-Biological Sciences 280. 10.1098/rspb.2013.0862 PMC3712418 23782882

[bib52] Sandler G. Beaudry F. E. G. Barrett S. C. H. Wright S. I., 2018 The effects of haploid selection on Y chromosome evolution in two closely related dioecious plants. Evolution Letters 197: 865. 10.1002/evl3.60 PMC6121804 30283688

[bib53] Schäfer A. M. Kemler M. Bauer R. Begerow D., 2010 The illustrated life cycle of *Microbotryum* on the host plant *Silene latifolia*. Botany-Botanique 88: 875–885. 10.1139/B10-061

[bib54] Scott M. F. Otto S. P., 2017 Haploid selection favors suppressed recombination between sex chromosomes despite causing biased sex ratios. Genetics 207: 1631–1649. 29051194 10.1534/genetics.117.300062 PMC5714470

[bib55] Seitner D. Uhse S. Gallei M. Djamei A., 2018 The core effector Cce1 is required for early infection of maize by Ustilago maydis. Mol. Plant Pathol. 19: 2277–2287. 10.1111/mpp.12698 29745456 PMC6638113

[bib56] Stamatakis A., 2006 RAxML-VI-HPC: Maximum Likelihood-based Phylogenetic Analyses with Thousands of Taxa and Mixed Models. Bioinformatics 22: 2688–2690. 10.1093/bioinformatics/btl446 16928733

[bib57] Stoletzki N. Eyre-Walker A., 2011 The positive correlation between dN/dS and dS in mammals is due to runs of adjacent substitutions. Mol. Biol. Evol. 28: 1371–1380. 10.1093/molbev/msq320 21115654

[bib58] Thomas A. Shykoff J. Jonot O. Giraud T., 2003 Sex-ratio bias in populations of the phytopathogenic fungus *Microbotryum violaceum* from several host species. Int. J. Plant Sci. 164: 641–647. 10.1086/375374

[bib59] Thrall P. H. Jarosz A. M., 1994 Host-pathogen dynamics in experimental populations of *Silene alba* and *Ustilago violacea*. 1. experimental tests of theoretical models. J. Ecol. 82: 561–570. 10.2307/2261264

[bib60] Uyenoyama M. K., 2005 Evolution under tight linkage to mating type. New Phytol. 165: 63–70. 10.1111/j.1469-8137.2004.01246.x 15720621

[bib61] Vercken E. Fontaine M. Gladieux P. Hood M. Jonot O., 2010 Glacial refugia in pathogens: European genetic structure of anther smut pathogens on *Silene latifolia* and *S. dioica*. PLoS Pathog. 6: e1001229 10.1371/journal.ppat.1001229 21187901 PMC3002987

[bib62] Villanueva-Cañas J. Laurie S. Albà M., 2013 Improving genome-wide scans of positive selection by using protein isoforms of similar length. Genome Biol. Evol. 5: 457–467. 10.1093/gbe/evt017 23377868 PMC3590775

[bib63] Wilch G. Ward S. Castle A., 1992 Transmission of mitochondrial DNA in *Ustilago violacea*. Curr. Genet. 22: 135–140. 10.1007/BF00351473 1358468

[bib64] Wright A. Dean R. Zimmer F. Mank J., 2016 How to make a sex chromosome. Nat. Commun. 7: 12087 10.1038/ncomms12087 27373494 PMC4932193

[bib65] Xu J. P., 2005 The inheritance of organelle genes and genomes: patterns and mechanisms. Genome Biol. Evol. 48: 951–958. 10.1139/g05-082 16391664

[bib66] Yang Z., 2007 PAML 4: phylogenetic analysis by maximum likelihood. Mol. Biol. Evol. 24: 1586–1591. 10.1093/molbev/msm088 17483113

[bib67] Yang Z. H. Nielsen R., 2000 Estimating synonymous and nonsynonymous substitution rates under realistic evolutionary models. Mol. Biol. Evol. 17: 32–43. 10.1093/oxfordjournals.molbev.a026236 10666704

[bib68] Zhang M. Feng H. Zhao Y. H. Song L. L. Gao C., 2018 *Valsa mali* pathogenic effector VmPxE1 contributes to full virulence and interacts with the host peroxidase MdAPX1 as a potential target. Front. Microbiol. 9: 11. 29922244 10.3389/fmicb.2018.00821 PMC5996921

[bib69] Zillig H., 1921 Über spezialisierte Formen beim Antherenbrand, *Ustilago violacea* (Pers.) Fuck. Zentralbl. Bakteriol. 53: 33–74.

